# Durable Objective Response to Lurbinectedin in Small Cell Bladder Cancer with TP53 Mutation: A Molecular-Directed Strategy

**DOI:** 10.3390/curroncol31060254

**Published:** 2024-06-13

**Authors:** Mohammad Jad Moussa, Jaanki Khandelwal, Nathaniel R. Wilson, Sagar A. Naik, Vivek Subbiah, Matthew T. Campbell, Pavlos Msaouel, Parminder Singh, Omar Alhalabi

**Affiliations:** 1 Department of Genitourinary Medical Oncology, Division of Cancer Medicine, University of Texas MD Anderson Cancer Center, Houston, TX 77030, USA; mmoussa1@mdanderson.org (M.J.M.);; 2 Department of Internal Medicine, McGovern Medical School, University of Texas Health Sciences Center at Houston, Houston, TX 77030, USA; 3 Division of Hematology and Oncology, Department of Internal Medicine, University of Michigan, Ann Arbor, MI 48109, USA; 4 Department of Abdominal Imaging, Division of Diagnostic Imaging, University of Texas MD Anderson Cancer Center, Houston, TX 77030, USA; 5 Department of Cancer Medicine, Sarah Cannon Research Institute, Nashville, TN 37203, USA; 6 Department of Internal Medicine, Division of Hematology and Medical Oncology, Mayo Clinic, Phoenix, AZ 85054, USA

**Keywords:** lurbinectedin, small cell bladder cancer, neuroendocrine carcinoma of the bladder, targeted therapy, urothelial carcinoma, next-generation sequencing, transcription factors

## Abstract

Small cell bladder cancer (SCBC) is a rare and aggressive disease, often treated with platinum/etoposide-based chemotherapy. Key molecular drivers include the inactivation of onco-suppressor genes (*TP53*, *RB1*) and amplifications in proto-oncogenes (*MYC*). We report a patient with SCBC who achieved an objective and prolonged response to lurbinectedin, which has been approved for metastatic small cell lung cancer, after developing disease progression on cisplatin/etoposide and nivolumab/ipilimumab. A genomic analysis of a metastatic biopsy prior to lurbinectedin initiation revealed a *TP53* mutation and amplification of the cell cycle regulators *E2F3* and *MYCL*. A repeat biopsy following the development of lurbinectedin resistance showed a new actionable ERBB2 alteration without significant change in the tumor mutation burden (six mutations/Mb). The present report suggests that lurbinectedin may be active and should be further explored in SCBC harboring *TP53* mutations and amplifications in E2F3 and MYC family complexes.

## 1. Introduction

Small cell bladder cancer (SCBC) is a highly aggressive, poorly differentiated neuroendocrine neoplasm with very limited treatment options [1]. SCBC usually arises from urothelial neoplasms that are frequently admixed with a conventional urothelial carcinoma (UC) or other histological components. Traditionally, systemic treatment in UC includes platinum-based chemotherapy, anti-programmed cell death protein (PD-1), or anti-programmed cell death ligand 1 (PD-L1) immune checkpoint inhibitors (ICIs), and, more recently, antibody–drug conjugates like enfortumab vedotin (anti-Nectin-4) or sacituzumab govitecan (anti-Trop-2) [2]. However, therapies or drugs specifically targeting the small cell component remain less defined. Effective reported regimens include etoposide plus cisplatin (EP) or alternating regimens of ifosfamide plus doxorubicin and EP (IA/EP) [3,4]. Despite a highly chemo-sensitive biology, these tumors frequently relapse, with unsatisfactory survival outcomes of between 5 and 13 months being reported in large case series [3,4]. At the molecular level, SCBC universally harbors bi-allelic losses of *TP53* and *RB1*, but also shows frequent alterations in other cell cycle-related genes like *MDM2* and *CDKN2A*, *TERT* promoters, and epigenetic modifiers like *ARID1A* and *KDM6A* [1]. The optimal management of these tumors, especially in metastatic disease, remains poorly defined, and a standardized treatment approach has not yet been developed.

Lurbinectedin was approved by the Food and Drug Administration (FDA) as a second-line therapy for small cell lung cancer (SCLC) following evidence of progression after frontline platinum-based chemotherapy in 2020 [5]. It replaced topotecan which used to be the only FDA-approved therapy in the same treatment line around 16 years ago [6]. It has an acceptable safety profile and a less frequent administration schedule (once every twenty-one days) than that of topotecan (daily infusion for five consecutive days within a twenty-one-day cycle) [5]. The cost of a single 4 mg dose of a branded intravenous powder for injection is approximately USD 8831 [7]. Given the clinical resemblance between SCBC and SCLC, it is unknown whether this therapeutic strategy could be replicated in SCBC.

Herein, we present a patient with SCBC who was treated with lurbinectedin and achieved a prolonged objective response after progression on frontline EP and second line immunotherapy. The decision to initiate lurbinectedin treatment was guided by the observed somatic alterations identified in tumoral biospecimens prior to treatment.

## 2. Case Description

A 71-year-old female, with a past medical history of obstructive sleep apnea and former smoking, initially presented to her primary care physician with lower urinary tract symptoms consisting of increased urinary frequency, urgency, and hematuria. She was referred to a gynecologist who found uterine fibroids on imaging and performed a dilation and curettage, which did not relieve the lower urinary tract symptoms. After she presented to a urologist who performed a cystoscopy that revealed a bladder tumor, she was then referred to our center. A transurethral resection of the tumor revealed urothelial carcinoma (UC) with high grade neuroendocrine differentiation. Initial staging scans showed metastatic disease in the cervical spine, sacrum, lungs, liver, and pelvic lymph nodes. Despite a significant response to six cycles of frontline EP, restaging 3 months after the end of treatment showed disease progression in the lungs and bladder.

A biopsy of a lung lesion confirmed small cell carcinoma histology and was analyzed by our MD Anderson Mutation Analysis Precision Panel using a targeted sequencing-based assay (*n* = 610 genes). Notable findings included amplifications in the cell cycle regulators *E2F3* and *MYCL* (Table 1) and the loss of a functional *TP53* mutation (Table 2). No genetic fusions were found; the tumor mutational burden (TMB) was low (five mutations/MB); and the microsatellite status was stable. Using clone 22C3 for PD-L1 staining, the combined positive score (CPS) was less than 1%.

**Figure 1:**
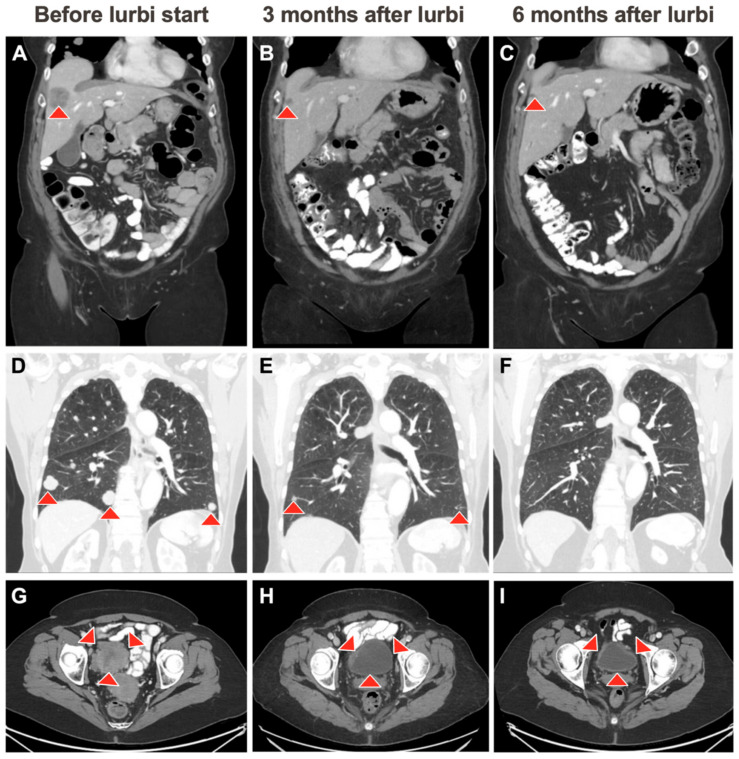
Radiologic evidence of tumor responses to lurbi (lurbinectedin). Images (**A**,**D**,**G**) demonstrate the tumor burden in the liver, lungs, and pelvis, respectively, prior to starting lurbinectedin. Images (**B**,**E**,**H**) were obtained 3 months after lurbinectedin initiation and demonstrate decrease in hepatic and pulmonary metastasis and decrease in pelvic adenopathy. Images (**C**,**F**,**I**) show further reduction in hepatic and pulmonary metastasis and pelvic adenopathy after 6 months of lurbinectedin treatment.

The patient was started on a second-line nivolumab plus ipilimumab therapy (nivo/ipi) as part of a clinical trial. After increasing pelvic pain and occasional hematuria, expedited restaging after four cycles of therapy indicated a lack of response. The case was then discussed with the rest of our faculty at the center of targeted therapy, who recommended the initiation of lurbinectedin, based on the patient’s mutational profile indicating the amplification of key cell cycle transcription factors. The first restaging imaging after the initiation of lurbinectedin treatment demonstrated a significant decrease in the size of the primary bladder tumor, metastatic hepatic lesions, peritoneal implants, thoracic, and pelvic adenopathy, as well as a near regression of several lung nodules (Figure 1). Restaging after seven cycles of therapy showed partial response and a significant reduction in metastatic lesions in the lung, liver, and pelvic lymph nodes. During this encounter, she reported a major improvement in her urinary symptoms, and denied experiencing dysuria, hematuria, urinary frequency, or abdominal pain.

Overall, the treatment was well tolerated. According to the Common Terminology Criteria for Adverse Events version (CTCAE) 5.0 criteria, one notable treatment-related adverse event (TRAE) included a four-fold aspartate aminotransferase [AST] elevation (Grade 2, AST: 122 U/L [N: ≤32]) and a six-fold alanine aminotransferase [ALT] elevation (Grade 3, ALT: 198 U/L [N: ≤33]), despite previous normal AST/ALT levels in the context of her hepatic metastasis. Other less serious TRAEs included Grade 1 anemia (Hemoglobin [Hgb] lowest attained level: 10.3 g/dL] and Grade 1 fatigue. Her LDH levels were already elevated at 288 U/L [N: 135–314] and her bilirubin levels were within normal levels, without biliary dilation on imaging. Transaminitis was resolved at follow-up without any therapeutic interventions or dose reductions.

**Figure 2:**
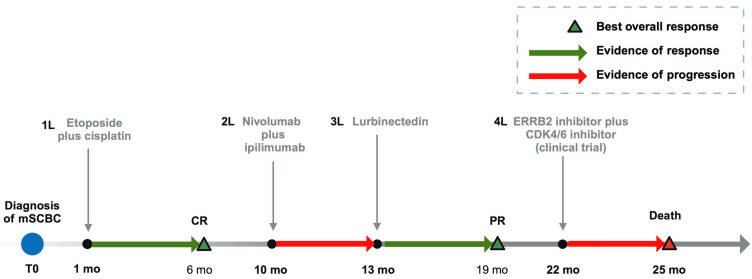
Timeline of main therapeutic events in the clinical trajectory of our patient. Abbreviations: mSCBC: Metastatic small cell bladder cancer; PR: partial response; mo: months.

Restaging after cycle 12 showed a progression of disease. A repeat biopsy of another lung tumor confirmed progression and its molecular findings are summarized in Table 1 and Table 2, including an actionable *ERBB2* alteration to S310F. The TMB of this specimen did not change significantly (six mutations/Mb) from that analyzed before the use of lurbinectedin. The patient was subsequently referred to a Phase 1 trial of ERBB2 inhibitor plus CDK4/6 inhibitor, and did not show a response upon restaging at 3 months. Unfortunately, the patient passed away four months after the last lurbinectedin dose. Her therapeutic timeline is shown in Figure 2.

## 3. Discussion

Lurbinectedin was used off-label in our case, as the choice of therapies in metastatic SCBC after progression on platinum-based chemotherapy is very limited. For example, limited data from patients with SCBC support nivo/ipi, which was unsuccessful for our patient [8]. Moreover, despite the advent of enfortumab vedotin and sacituzumab govitecan in the treatment of metastatic UC [2], their activity in SCBC is expected to be low due to the lack of Nectin-4 and absent to low Trop-2 surface expression [9,10,11,12]. Moreover, radiation therapy was not employed because our patient presented with de novo multivisceral metastatic disease that required systemic treatment rather than targeted or localized approaches to address the disease burden.

**Figure 3:**
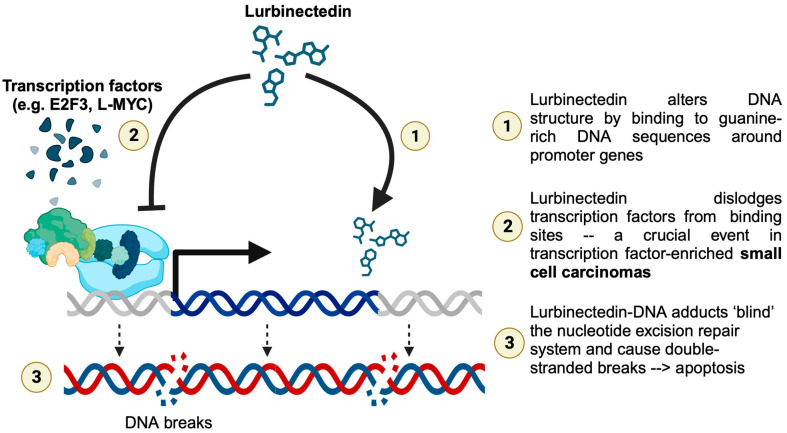
Our hypothesis about lurbinectedin’s action in our case of small cell bladder cancer, based on the correlation between our genetic and clinical results and the established mechanism of action of the drug in other diseases.

There is a growing interest in lurbinectedin combinations in the treatment of ovarian and endometrial cancer, soft tissue sarcomas, and pretreated neuroendocrine tumors [13]. Notably, lurbinectedin has shown activity in relapsed Ewing’s sarcoma, a round blue-cell tumor with similar immunohistochemical markers as small cell carcinoma [14]. The drug is a synthetic alkaloid that covalently binds to DNA, generating double-stranded breaks, disrupting DNA-protein interactions, and inhibiting RNA transcription [5] (Figure 3). It also induces apoptosis of tumor-associated macrophages and decreases chemokine production. An earlier report described a complete metabolic response to lurbinectedin on a PET-CT scan after chemotherapy and radiation therapy in a 70-year-old patient with SCBC [15]. The authors postulated that lurbinectedin acted by inhibiting RNA polymerase II, which is commonly hyperactive in SCLC. 

Our patient experienced one ≥Grade 3 TRAE (ALT elevation), which was, however, resolved without the needed for an intervention or dose reduction. Grade 1 anemia was also consistent with previous reports—transient and reversible myelotoxicity was manageable in the largest pooled analysis of the drug [16]. The most frequent Grade 3 or higher TRAEs were also mostly hematological, including neutropenia (41%), leukopenia (30%), anemia (17%), and thrombocytopenia (10%), while the most frequent non-hematological TRAEs were fatigue (53%), or nonspecific gastrointestinal disturbances [nausea (51%), vomiting (25%), constipation (17%), and diarrhea (13%)] [16,17]. Well tolerated in elderly patients (≥65 years), a population with higher incidence of advanced bladder cancer, the drug did not entail major dose modifications in most treated patients (79%) [16].

While metastases from SCLC to the bladder are described in the literature [18], several lines of evidence suggest that the present case is a primary SCBC with pulmonary metastasis. First, the genomic profile of the metastatic tumor is strongly in favor of a urothelial origin. The *TERT* c.-124C>T mutation, observed with a high variant allele frequency (VAF) of 50% even before lurbinectedin treatment in our case [19], is commonly associated with bladder cancer. Furthermore, the clinical picture with no dominant malignant lung nodule is also consistent with primary bladder cancer. Of note, *E2F3* amplification, a frequent event in UC, and *ERBB2* S310F mutation, mostly prevalent in UC (at a rate of 3.64%), further suggest an aggressive urothelial origin as opposed to SCLC being metastatic in the bladder [20,21,22].

In a single-arm phase II trial in patients with SCLC progressing on systemic chemotherapy (*n* = 105), lurbinectedin showed a median overall survival (OS) of 9.3 months (95% CI: 6.3–11.8), a median progression-free survival of 3.5 months (95% CI: 2.6–4.3), and an overall response rate of 35.2% [23]. In June 2020, lurbinectedin was granted accelerated approval as a second-line agent in patients with SCLC. In the phase III ATLANTIS trial, lurbinectedin plus doxorubicin did not improve OS compared to the investigator’s choice of chemotherapy. However, it did have a more favorable hematological safety profile than the control group, as the most common grade ≥3 TRAEs of the combination consisted of fatigue and GI symptoms [24]. 

In our case, the tumor harbored mutations in the tumor suppressor *TP53*, and amplifications in the cell cycle regulators *E2F3* and *MYCL*. MYC proteins, or “master gene regulators”, regulate the genes involved in cell growth, the cell cycle, differentiation, apoptosis, DNA repair, and protein translation [25]. They also control transcription mediated by all three RNA polymerases. *E2F3* amplification may represent a lineage-specific event in bladder cancer because amplification of this region rarely occurs in other epithelial tumor types (21% vs. 4.9% of 1932 nonurothelial epithelial tumors) [26]. Both *E2F3* amplification and *RB1* deletion/mutation were more prevalent in the subset of tumors exhibiting neuroendocrine differentiation compared with those with a predominantly urothelial morphology (*E2F3*: 50% vs. 17%, *p* = 0.03; *RB1*: 50% vs. 13%, *p* = 0.01) [27]. Given the role of lurbinectedin in inhibiting oncogenic transcription and DNA repair machinery in tumor cells, it is worth investigating the impact of different related genomic alterations on the response to this drug in bladder cancer.

Early reports about the activity of lurbinectedin in metastatic neuroendocrine carcinomas (mNEC) of the genitourinary tract are emerging. The overall response rate (ORR) was 42.8% in a small cohort (*n* = 7) of neuroendocrine carcinomas of the bladder, and the median duration of response was 7.4 months (95% CI: 2.3–9.5) for four patients with responding tumors (three with bladder mNEC and one with prostate mNEC) [28]. In another cohort of patients with prostatic NEC (*n* = 16 response-evaluable patients), the objective response rate (ORR) was 31.3%, while the median OS and progression-free survival (PFS) from the first lurbinectedin dose were 6 months (95% CI: 0.2–16.7) and 3.3 months (95% CI: 0.2–7.8), respectively [29]. Recently, a phase II, open-label, nonrandomized study (LASER) [NCT06228066] of lurbinectedin, with and without avelumab, has started enrolling patients with SCBC or other high-grade neuroendocrine tumors of the urinary tract [30]. The study is designed with two cohorts: Cohort 1 enrolls participants with prior ICI exposure or ineligible to receive them, while Cohort 2 enrolls ICI-naïve participants eligible to receive them [31]. Patients in Cohort 1 will receive single-agent lurbinectedin 3.2 mg/m^2^ IV every 21 days, while patients in Cohort 2 will receive lurbinectedin 3.2 mg/m^2^ IV and avelumab 800 mg IV every 21 days. With the rarity of SCBC diagnosis, multi-institutional collaborations and translational research are warranted to study the biology behind this drug in the treatment of SCBC and other neuroendocrine carcinomas.

## Figures and Tables

**Table 1:** Copy Number Variations (CNVs) prior to and after lurbinectedin therapy.

Genes, Prior to Lurbinectedin Treatment	Genes, after Lurbinectedin Treatment	Finding	Cytoband
*E2F3*	*E2F3*	Amplification	6p22.3
*ERG*	*ERG*	Amplification	21q22.2
*FOXA1*	*-*	Amplification	14q21.1
*MYCL*	*MYCL*	Amplification	1p34.2
*SDHC*	*SDHC*	Amplification	1q23.3
*-*	*SOX10*	Amplification	22q13.1

**Table 2:** Mutational profile, including sequence variants and single nucleotide variants (SNVs), prior to and after lurbinectedin therapy. VAF: Variant Allele Frequency.

Genes, Prior to Lurbinectedin Treatment	Genes, after Lurbinectedin Treatment	DNA	Protein	Location	VAF, Prior to Lurbinectedin Treatment	VAF, after Lurbinectedin Treatment	Type
*MGA*	*MGA*	c 8014C>T	p. P2672S	Exon 24	23%	31%	SNV—Missense
*STAT5B*	*STAT5B*	c 1165C>T	p. R389C	Exon 9	17%	42%	SNV—Missense
*STK19*	*STK19*	c 97G>T	p. E33	Exon 1	28%	20%	SNV—Nonsense
*TERT*	*TERT*	c 124C>T	-	UTR5	50%	42%	SNV
*TP53*	*TP53*	c 192del	p. R65fs*58	Exon 4	76%	84%	Deletion—Frameshift
*-*	*AMER1*	c 533G>A	p. R176H	Exon 2	-	27%	SNV—Missense
*-*	*ELF3*	c 218T>C	p. L73P	Exon 3	-	21%	SNV—Missense
*-*	*ERBB2*	c. 929C>T	p. S310F	Exon 8	-	21%	SNV—Missense
*-*	*FLT1*	c 2306C>T	p. A769V	Exon 16	-	23%	SNV—Missense
*-*	*IRS2*	C 994C>A	p. P332T	Exon 1	-	22%	SNV—Missense

## Data Availability

No new data were created or analyzed in this study. Data sharing is not applicable to this article.
